# The Principle of Inverse Effectiveness in Audiovisual Speech Perception

**DOI:** 10.3389/fnhum.2019.00335

**Published:** 2019-09-26

**Authors:** Luuk P. H. van de Rijt, Anja Roye, Emmanuel A. M. Mylanus, A. John van Opstal, Marc M. van Wanrooij

**Affiliations:** ^1^Department of Otorhinolaryngology, Donders Institute for Brain, Cognition, and Behaviour, Radboud University Medical Center, Nijmegen, Netherlands; ^2^Department of Biophysics, Donders Institute for Brain, Cognition, and Behaviour, Radboud University, Nijmegen, Netherlands

**Keywords:** multisensory, lipreading, listening, hearing, speech recognition in noise

## Abstract

We assessed how synchronous speech listening and lipreading affects speech recognition in acoustic noise. In simple audiovisual perceptual tasks, inverse effectiveness is often observed, which holds that the weaker the unimodal stimuli, or the poorer their signal-to-noise ratio, the stronger the audiovisual benefit. So far, however, inverse effectiveness has not been demonstrated for complex audiovisual speech stimuli. Here we assess whether this multisensory integration effect can also be observed for the recognizability of spoken words. To that end, we presented audiovisual sentences to 18 native-Dutch normal-hearing participants, who had to identify the spoken words from a finite list. Speech-recognition performance was determined for auditory-only, visual-only (lipreading), and auditory-visual conditions. To modulate acoustic task difficulty, we systematically varied the auditory signal-to-noise ratio. In line with a commonly observed multisensory enhancement on speech recognition, audiovisual words were more easily recognized than auditory-only words (recognition thresholds of −15 and −12 dB, respectively). We here show that the difficulty of recognizing a particular word, either acoustically or visually, determines the occurrence of inverse effectiveness in audiovisual word integration. Thus, words that are better heard or recognized through lipreading, benefit less from bimodal presentation. Audiovisual performance at the lowest acoustic signal-to-noise ratios (45%) fell below the visual recognition rates (60%), reflecting an actual deterioration of lipreading in the presence of excessive acoustic noise. This suggests that the brain may adopt a strategy in which attention has to be divided between listening and lipreading.

## Introduction

Speech is a complex, dynamic multisensory stimulus, characterized by both an auditory and a visual information stream. Congruent information of the sensory modalities (i.e., spatial and temporal coincidence of the sensory streams, and their meanings) is integrated in the brain (Calvert et al., 2000; van de Rijt et al., 2016) to form a coherent, often enhanced, percept of the common underlying source (Stein and Meredith, 1993). Indeed, additional synchronous visual information (i.e., speech-reading/lipreading) has a positive impact on speech perception, and audiovisual speech recognition in acoustic noise is substantially better than for auditory speech alone (O’Neill, 1954; Sumby and Pollack, 1954; MacLeod and Summerfield, 1987, 1990; Helfer, 1997; Grant and Seitz, 2000; Bernstein et al., 2004; Sommers et al., 2005; Ross et al., 2007; Tye-Murray et al., 2007, 2010; Winn et al., 2013).

Audiovisual integration in general, has been the topic of a variety of behavioral and electrophysiological studies, involving rapid eye-orienting to simple peripheral stimuli (Corneil et al., 2002; van Barneveld and van Wanrooij, 2013), spatial and temporal discrimination of audiovisual objects (Alais and Burr, 2004; Wallace et al., 2004; Körding et al., 2007), and the integrative responses of single neurons in cats and monkeys (Meredith and Stein, 1986; Wallace et al., 1998; Bell et al., 2005). Three main principles have been shown to govern the mechanisms of multisensory integration: (i) spatial alignment of the different sources, (ii) temporal (near-)synchrony, and (iii) inverse effectiveness. The latter holds that multisensory enhancement strongly increases for poorly perceptible unisensory signals, for example in the presence of acoustic background noise or visual distracters (Stein and Meredith, 1993). Although these principles have mostly been demonstrated at the neurophysiological level of anesthetized experimental animals (for review, see Stein and Meredith, 1993), several studies on audiovisual saccadic eye movements in humans or on manual reaction times in macaques and humans (Bremen et al., 2017), have revealed systematic modulations of the effects of audiovisual congruency and inverse effectiveness that corroborate the neurophysiological data (Frens et al., 1995; Corneil et al., 2002; van Wanrooij et al., 2009).

In this study, we focus on whether the phenomenon of inverse effectiveness can also be applied to speech perception. This is not a trivial extension of the classical audiovisual integration studies, as the underlying speech-related sensory signals are complex and dynamic signals, requiring advanced (top–down) neural processing within the auditory and visual systems. One way of studying the presence of inverse effectiveness in the perception of audiovisual speech stimuli is by adding background noise (Ross et al., 2007; Ma et al., 2009; Tye-Murray et al., 2010), which effectively changes the saliency of the auditory stimulus. By doing so, earlier studies have suggested an absence of inverse effectiveness, as at low unimodal performance scores, the audiovisual enhancement decreases. The principle of inverse effectiveness has also been studied by quantifying the differences in unimodal word-recognition performance scores across (groups of) subjects (Rouger et al., 2007; Tye-Murray et al., 2010, 2016; Winn et al., 2013), however, outcomes were not consistent. To our knowledge, the effect of the visual or auditory recognizability of words (irrespective of background noise) on the presence or absence of inverse effectiveness has not been studied. For example, words that contain more spectral-temporal information, or are articulated more pronouncedly, will likely be better heard or visually recognized over a large range of noise levels. If the principle of inverse effectiveness would hold at the word level, highly informative words should benefit less from bimodal presentation than less-informative words. To study this possibility, we determined how well words can be recognized by listening and/or lipreading under noisy listening conditions in normal-hearing subjects.

## Results

### Overview

**FIGURE 1:**
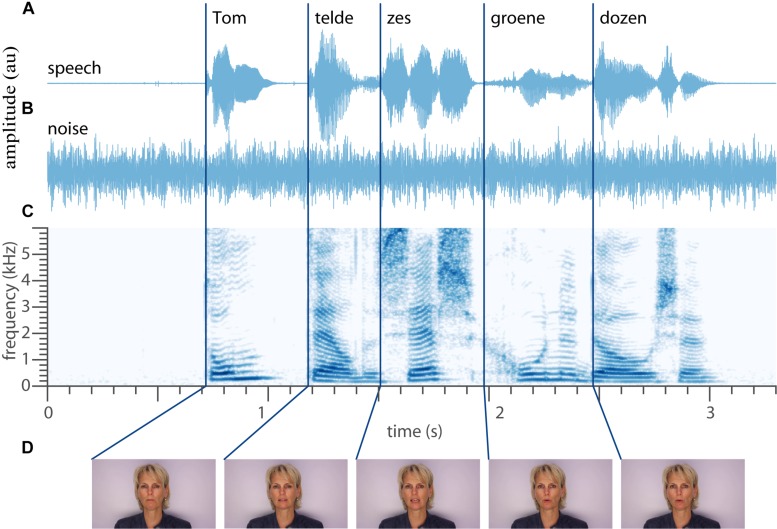
Example stimulus. **(A)** Temporal waveform of the auditory speech signal “Tom telde zes groene dozen” (translation: Tom counted six green boxes.) **(B)** Waveform of the auditory noise. **(C)** Spectrogram of the recorded sentence. **(D)** Five videos frames around the onset of the word. Dark blue lines denote the approximate onset of each individual word. Written informed consent for the publication of this image was obtained from the individual shown.

Eighteen normal-hearing subjects had to identify 50 words (Table 1) occurring in 155 unique five-word sentences, by selecting the words they recognized (10-alternative forced choice) on a screen. The speech material was based on the Dutch version of the speech-in-noise matrix test developed by Houben et al. (2014; see section Materials and Methods on the construction of the speech material, Figure 1). The words were presented in acoustically only (A-only, e.g., Figure 1A), visual-only (V-only, e.g., Figure 1D) or bimodal (AV, e.g., Figures 1A,D combined) blocks. An acoustic background noise (Figure 1B) was played in the A-only and AV conditions at five signal-to-noise ratios. Note that the words vary substantially in ongoing amplitude and duration (Figure 1A), spectral-temporal dynamics (Figure 1C), and articulation (Figure 1D). This variation will likely affect speech recognition, and is the foundation on which we will test inverse effectiveness. In what follows, we will quantify how well each word is recognized visually and aurally, then how simultaneous audiovisual presentation of a word affects recognition accuracy, and finally we will determine how unimodal recognition accuracy affects audiovisual enhancement.

**TABLE 1:** Words of the Dutch matrix test.

**Name**	**Verb**	**Numeral**	**Adjective**	**Object**
Anneke	geeft	twee	dure	bloemen
Christien	had	drie	goede	boeken
Heleen	kiest	vier	**groene**	boten
Jan	koopt	vijf	grote	**dozen**
Mark	maakte	**zes**	kleine	fietsen
Monique	tekent	acht	mooie	messen
Pieter	**telde**	negen	nieuwe	munten
Sarah	vond	tien	oranje	ringen
**Tom**	vroeg	twaalf	vuile	schoenen
Willem	wint	achttien	zware	stenen

*Bold words indicate an example sentence: ‘Tom telde zes groene dozen’ (translation: ‘Tom counted six green boxes’, see Figure 8).*

### Lipreading

**FIGURE 2:**
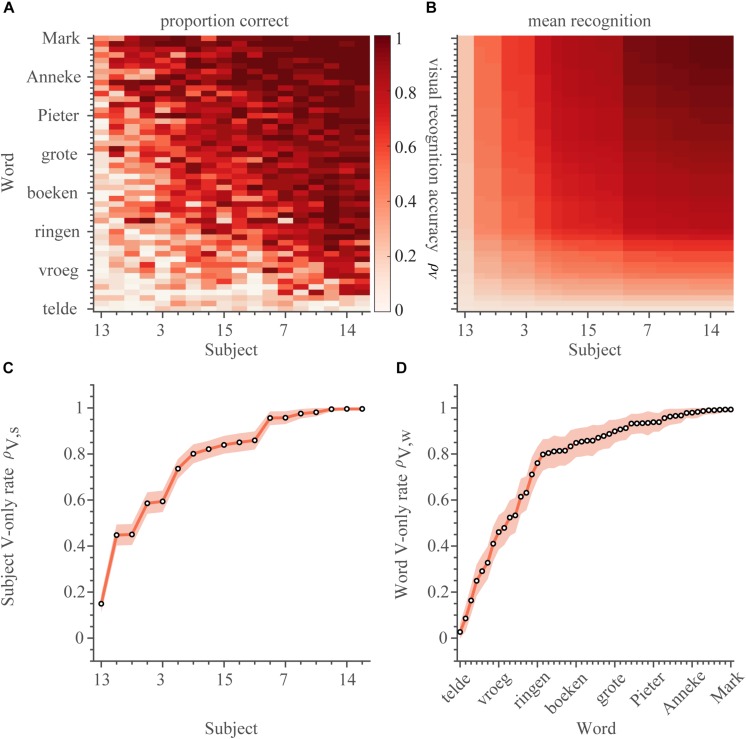
Lipreading. **(A)** Visual recognition scores. The correct score (number of correct responses divided by the number of presentations) is shown separately for every word and subject (900 entries) for the V-only condition. The correct scores and rates have been ordered by the recognition rates of subjects on the abscissa, and of words on the ordinate from low-left to high-right. **(B)** The average estimated visual recognition rates (Equation 1). Same layout as in **(A)**. V-only speech recognition rates for **(C)** subjects and **(D)** words. Rates were ordered from low-left to high-right. Open circles indicate the mean of the estimated rate, colored patch indicates the 95% Highest Density Interval (HDI). Reddish colors denote visual conditions.

We will first describe the lipreading abilities (V-only). These were quantified for every subject (*n* = 18) and every word (*n* = 50) as the number of correct responses, z, divided by the number of presentations, (*N* = 18), i.e., the correct scores (Figure 2A), in the V-only block. The correct scores varied both across words and subjects from perfect (i.e., 18 correct responses to 18 presentations, e.g., for the word ‘vijf’ by subject S2), to around chance level (0.1, e.g., a score of 0 correct responses for 18 word presentations for the word ‘telde’ presented to subject S8). Notably, some words were easily correctly identified by almost all subjects (e.g., ‘Mark’), while others were near-never identified (‘telde’) by anyone. Similarly, some subjects were perfect lip-readers with correct scores for all words near 1.0 (e.g., subject S14), while subject S13, as an extreme case, could hardly identify any words via lipreading.

As the realizations of the visual correct scores were quite noisy (as apparent in the jittery pattern in Figure 2A), the estimates for the proportion of correct scores for each word and subject separately were quite uncertain (average 95%-highest density interval [95%-HDI] was 0.29 [0.14–0.42] across all 900 estimates from 18 subjects and 50 words). We therefore determined the visual lipreading recognition rates for words, ρ_V,w_, and for each subject, ρ_V,s_ by fitting the following function:

(1) $${\text{F}}_{V}\,\left(\rho_{V,w},\rho_{V,s}\right)=\rho_{V,w}\times \rho_{V,s}$$

to the responses from the V-only trials, which are taken to be binomially distributed (see Materials and Methods for details on the fitting procedure). This yields 18 visual recognition rates for subjects, *ρ_*V,s*_*, and 50 visual recognition rates for words, *ρ_*V,w*_*. Multiplication of these rates assumes that they were independent, and thus separable from each other. This assumption seems to hold, at least qualitatively, when looking at the correct scores for each word and subject (cf. Figures 2A,B, see also section Materials and Methods for a more quantitative approach). This procedure smoothened the recognition rate matrix (Figure 2B), and decreased variability in the estimates (as expressed by the small 95%-HDI in Figures 2C,D; average 95%-HDI = 0.09 [0.04–0.14] across 68 parameters). This function also reduced the number of variables from 900 (number of subjects multiplied by number of words) to 68 (number of subjects plus number of words). These features enable a more practical comparison to the other, A-only and AV conditions, to be introduced later on. The model described by equation 1 is also preferred by having a lower Bayesian Information Criterion (BIC, see section Materials and Methods) compared to the model that determines recognition rates independently for all subjects and words (5.5 k vs. 9.0 k, respectively).

Moreover, the recognition estimates are in line with the correct-score data (correlation *r* = 0.84, with limited to no discernible bias). Words were generally easily recognized through lipreading (Figure 2D, mean *ρ_*V,w*_* = 0.77), but there was considerable variability in visual recognizability across words: many words were identified easily (e.g., mean *ρ_*V,boten*_* = 0.99), while others were barely recognizable (e.g., mean *ρ_*V,telde*_* = 0.03). Also the ability of subjects to lipread was relatively high on average (Figure 2C, mean *ρ_*V,s*_* = 0.78). However, there was a considerable range in lipreading ability. The best lip-readers could recognize ∼100% of the easily-identified words (mean *ρ_*V,S*__14_* = 1.00), while the worst performer could at best recognize ∼15% correctly (mean *ρ_*V,S*__13_* = 0.15). The large variability in visual recognition rates across words and subjects provides a potential way to determine how speech-reading performance affects speech listening, when both auditory and visual speech-recognition cues are presented synchronously.

### Speech Listening

**FIGURE 3:**
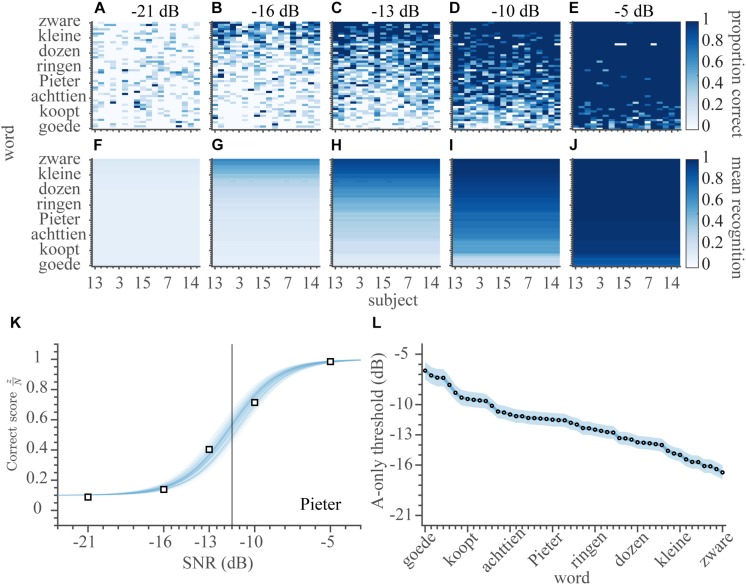
Speech listening. Auditory word-recognition scores. **(A–E)** The correct score (number of correct responses divided by the number of presentations) is shown separately for every word and subject (900 entries) for each of the SNRs of −21, −16, −13, −10, and −5 dB. The correct scores have been ordered by the average V-only rates of subjects on the abscissa, and A-only thresholds on the ordinate. **(F–J)** The average estimated auditory recognition rates. **(K)** Correct scores and psychometric fit for the word ‘Pieter’ as a function of SNR, averaged across all subjects. Open squares indicate the measured correct scores. Blue shading denotes credible fits (see Materials and Methods). Vertical bold gray line indicates the average of likely recognition thresholds. **(L)** A-only speech recognition thresholds, ordered from high-left to low-right. Note that a lower threshold indicates better performance. Open circles indicate means of the estimated thresholds, colored patch indicates the 95% HDI. Blueish colors denote auditory conditions.

In the A-only block, subjects identified words by listening to the audio recordings of sentences (e.g., Figure 1A, without visual feedback from the lips). A stationary masking noise (e.g., Figure 1B) was played at a constant level of 65 dB SPL, while the sentences were played at an SNR of −21, −16, −13, −10, or −5 dB. In total, the data comprised 4482 different combinations of subject, word, and SNR (not all 250 potential combinations of SNR and word were presented to every one of the 18 subjects). The average word recognition rate was ∼50% across all SNRs and subjects (Figures 3A–E). Overall listening performance for SNRs lower than −10 dB was worse than lipreading performance (cf. amount of white in Figure 2A vs. Figures 3A–E). In contrast to lipreading, listening performance was quite similar across subjects (Figures 3A–E). This small variability across listeners might be expected, as all listeners were normal-hearing, and were therefore likely to understand the speech equally well.

Typically, SNR had a strong influence on the ability to recognize the words through listening (Figures 3A–E, from low to high SNR, the correct scores improve from almost 0 to near perfect). To quantify this, we estimated the SNR for which the recognition rate was 50%, i.e., the auditory speech-recognition threshold, *θ_*A*_*, by fitting the parameters of a logistic psychometric function *F*_*A*_ for every word (with a parametrization as mentioned in Kuss et al., 2005):

(2) $${\text{F}}_{A}\left(SNR,{\text{θ}}_{A},{\text{ω}}_{A}\right)={\left(1+e^{-\frac{2In9}{{\text{ω}}_{A}}\left(SNR-{\text{θ}}_{A}\right)}\right)}^{-1}$$

with *ω_*A*_* the auditory recognition width from 10 to 90% performance (in dB). The width (conversely, the slope) of the psychometric curve, *ω_*A*_*, did not vary substantially across words or subjects. Therefore, only one value was estimated, which was on average 7.1 dB, 95% HDI: 6.8 – 7.4 dB. As the correct scores did not vary appreciably across subjects, we pooled over subjects, to obtain 50 auditory recognition thresholds, one for each word. To exemplify this, we take a look at the word ‘Pieter’ (Figure 3K). This word was easily recognized by all subjects at the SNR of −5 dB, leading to a 100% recognition score. In contrast, “Pieter” was almost impossible to identify at the lowest SNR of −21 dB, when subjects identified the word presented in 10% of the cases (chance-level). By fitting a psychometric curve through the data, we obtained a speech listening threshold for this word at −11.5 dB (Figure 3K). Similar to the V-only model (equation 1), this modeling smoothened the A-only estimates (Figures 3F–J), reduced uncertainty in the parameter estimates (average 95%-HDI from 0.54 [0.35–0.77] to 0.07 [0.00–0.18]), and reduced the number of parameters (from 4482 to 51). The model is (therefore) also favored by the BIC (8.0 k vs. 45.3 k of a fully independent model; a model that included a logistic dependence on SNR but allowed for subject and word variability in both the threshold and width had a BIC of 21.2 k with 1800 free parameters).

Importantly, auditory speech-recognition thresholds for each word (Figure 3L) varied over a considerable 10-dB range, from the best-recognizable word (mean *θ_*A,zware*_* = −16.7 dB) to the hardest-to-recognize word (mean *θ_*A,goede*_* = −6.6 dB), with an average threshold of −12.1 dB.

### Audiovisual Speech Recognition

**FIGURE 4:**
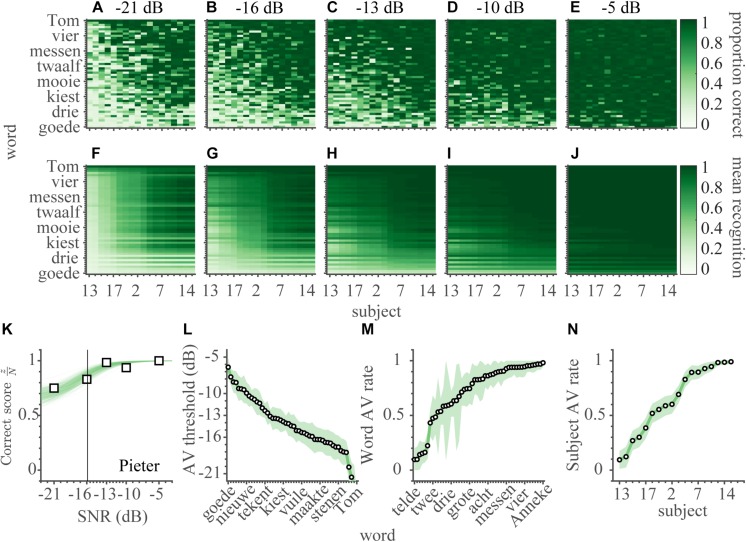
Audiovisual speech recognition. **(A–E)** The audiovisual correct scores are shown separately for every word and subject (900 entries) for each of the SNRs of **(A)** −21, **(B)** −16, **(C)** −13, **(D)** −10, and **(E)** −5 dB. The correct scores have been ordered by the average AV recognition rates of subjects on the abscissa, and of words on the ordinate. **(F–J)** The average estimated audiovisual recognition rates. **(K)** Audiovisual correct scores and psychometric fit for the word ‘Pieter’ as a function of SNR, averaged across all subjects. Open squares indicate the measured correct scores. Green shading denotes credible fits (see Materials and Methods). Vertical bold gray line indicates the average of likely recognition thresholds. **(L)** AV speech-recognition thresholds, **(M,N)** AV recognition rates for words and subjects, ordered from low-left to high-right. Note that a lower threshold indicates better performance. Open circles indicate means of the estimated thresholds, colored patch indicates the 95% HDI. Greenish colors denote audio-visual conditions.

In the AV-condition, subjects identified words by listening to, and by lipreading, the audiovisual recordings of sentences in the presence of acoustic noise (65 dB SPL, SNR: [−21, −16, −13, −10, −5] dB). The presentation of congruent visual feedback clearly aided recognition performance, as the correct scores (Figures 4A–E) were higher than for the A-only condition (cf. Figures 3A–E). Also, in contrast to the speech listening scores (cf. Figures 3A–E) and more in line with lipreading performance (Figure 2A), the AV scores not only varied over words, but also across subjects (which is visible in the pattern of correct scores in Figure 4A).

We quantified AV performance by fitting a function *F*_*AV*_ that combines the characteristics of Equations 1 and 2 for the unimodal performances:

(3) FA⁢V(SNR,θA⁢V,ωA⁢V,ρA⁢V,w,ρA⁢V,s)=(1-ρA⁢V,w×ρA⁢V,s)×(1+e-2⁢l⁢n⁢ 9ωA⁢V⁢(S⁢N⁢R-θA⁢V))-1+ρA⁢V,w×ρA⁢V,s

with the audiovisual recognition threshold, *θ_*AV*_* describing the logistic SNR dependence, and two audiovisual recognition rates *ρ_*AV,w*_* and *ρ_*AV,s*_*, defining the minimum performance level in the AV condition (i.e., for SNR = −∞) for words and subjects, respectively. Again, the word ‘Pieter’ is taken as an example to illustrate the fit (Figure 4K, cf. Figure 3K). In contrast to A-only recognition, even at the lowest SNR (−21 dB), this word was easily recognized by all subjects in 75% of the time.

Similar to the V-only and A-only models (equations 1 and 2), this modeling smoothened the AV-only estimates (Figures 4F–J), reduced uncertainty in the parameter estimates (average 95%-HDI from 0.55 [0.35–0.77] to 0.10 [0.00–0.22]), and reduced the number of parameters (from 4482 to 119). Again, the model is favored by the BIC (7.7 k vs. 45.2 k of a fully independent model; a model that included a logistic dependence on SNR but allowed for subject and word variability in both the threshold and width had a BIC of 33.1 k with 1868 free parameters).

Like for the A-only condition, one value of the width was estimated for all subjects and words (this width was on average 10.5 dB, 95% HDI: 9.5 – 11.4 dB). The audiovisual speech thresholds were determined for words alone (Figure 4L), in line with the auditory speech thresholds (Figure 3L). The thresholds varied over a ∼21 dB range (from mean *θ_*A, Tom*_* = −27.6 dB to mean *θ_*A,goede*_* = −6.4 dB), with an average threshold of −14.7 dB. The subjects’ AV recognition rates (Figure 4N) varied from almost negligible (chance) to near-perfect (from mean *ρ_*AV,S*__13_* = 0.07 to mean *ρ_*AV*,__*S*__14_* = 0.99), with an average rate around 0.63. The AV recognition rates for words (Figure 4M) varied over a similar range (from mean *ρ_*AV,tekent*_* = 0.09 to mean *ρ_*AV,Anneke*_* = 0.98), with an average rate around 0.71. There was considerable uncertainty in the estimation of the word AV rates (e.g., the widest 95%-HDI = 0.02–0.95 for the word ‘Tom’), but in general the 95% HDIs for all other parameters were narrow.

### Audiovisual Enhancement

The audiovisual parameters from equation 3 are basic descriptors for the audiovisual performance, from which we can derive the audiovisual enhancement by comparing the results to the unimodal parameters from equations 1 and 2. For the audiovisual threshold, the comparison to the auditory threshold indicates how much the SNR can decrease when the visual modality is added, without affecting performance. The change in threshold,Δ*θ_*AV*_*, relative to the auditory threshold, was thus estimated by rewriting *θ_*AV*_* in equation 3 as:

(4) $$\theta_{A\,V}=\theta_{A}+\Delta \,\theta_{A\,V}$$

**FIGURE 5:**
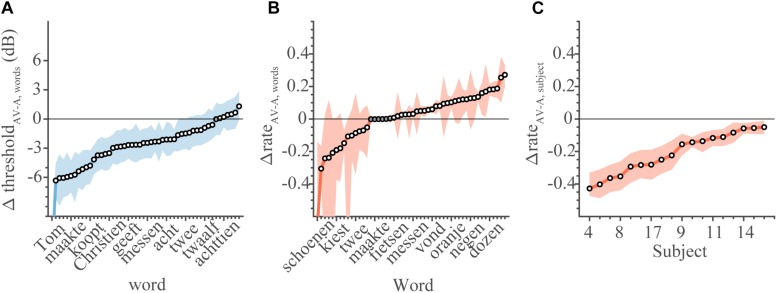
Comparison between audiovisual and unimodal conditions. Change in threshold and rates of AV speech recognition in comparison to unimodal listening conditions. **(A)** The change in threshold for each word (equation 4). Note that a negative change in threshold denotes better performance in AV conditions. **(B)** The change in recognition rate for each word (equation 5). **(C)** The change in recognition rate for each subject. For rates, a change larger than 0 denotes better AV performance. Open circles denote the mean of the parameter estimate, colored patches indicate 95% HDI.

Typically, the audiovisual recognition thresholds were lower (i.e., better) than the auditory recognition thresholds (Figure 5A), by on average −1.3 dB. This means that the threshold is typically reached at lower SNRs when people speech-read at the same time. The threshold for 35 words improved in the AV condition (95%-HDI lay below 0 dB), while for 15 words there was no difference (95%-HDI included 0 dB).

Similarly, the minimum performance level in the AV condition is given by multiplying the recognition rates for words and subjects:ρ_*AV,w*_×ρ_*AV,s*_. This measure quantifies the performance level in the absence of an auditory signal (i.e., when the SNR approaches −∞). In case there really is no auditory signal, one might expect that the minimum audiovisual performance level, given by the rates, would equal the visual performance rate. This, of course, only holds if the stimulus parameters fully determine the subject’s performance levels, and if non-stimulus factors, such as task or block design, are irrelevant. We tested this prediction by determining the difference in audiovisual and visual rates for words and subjects:

(5) $$\left\{ \begin{array}{c} \rho_{A\,V,w}=\rho_{V,w}+\Delta \,\rho_{A\,V,w}\\ \rho_{A\,V,s}{=}_{V,s}+\Delta \,\rho_{A\,V,s} \end{array}\right.$$

On average, there was no difference in recognition rates for words (Figure 5B), as the difference values scattered around 0 for most words. In contrast, the subjects’ ability to lipread in the AV condition (as reflected by the subjects’ recognition rate) was poorer than in the V-only condition (Figure 5C). The rates for all subjects dropped (mean Δ*ρ* = −0.2, all 95% HDI < 0). This indicates that, on average, audiovisual performance dropped below the V-only performance scores, when poor auditory SNRs caused speech listening to deteriorate completely.

As these last points are important, we will restate them. First, the AV threshold is lowered, making it easier to recognize words at a given SNR. This effectively yields an audiovisual enhancement to speech listening (Figure 5A). Second, words are recognized through lipreading at equal levels in both V-only and AV conditions (Figure 5B). Third, somewhat surprisingly, the lipreading ability of subjects is impoverished in the AV condition (Figure 5C). This suggests that task constraints (i.e., being in an AV condition vs. in a V-only condition) have a significant influence on speech recognition performance, even when stimulus parameters are equivalent (i.e., only a visual, no auditory signal).

### Probability Summation

**FIGURE 6:**
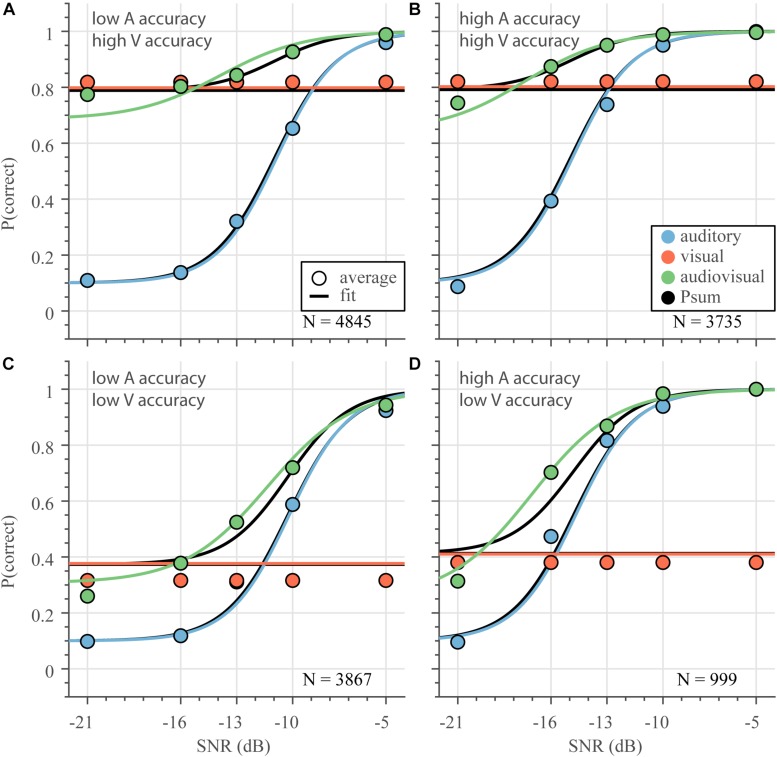
Audiovisual speech recognition varies with unimodal information. Psychometric curves were determined (equation 1–3) from all data divided across 4 groups differing in unimodal performances: visual recognition rate **(A,B)** larger and **(C,D)** smaller than 0.55**;** and an auditory recognition rate **(A,C)** larger than and **(B,D)** smaller than 0.55. Curves indicate the average model estimate, circles denote the average correct score. N is the number of subject-word-SNR combinations for each group.

Next, we qualitatively compared the AV condition with a model in which audiovisual integration is merely a result of statistical summation rather than of true neural integration. Finding an improved performance (i.e., better speech recognition) in the AV condition is not automatic evidence that the brain integrates the auditory and visual inputs. Indeed, having both modalities available, rather than one, automatically increases the probability of stimulus recognition. In a model of probability summation, participants recognize a word from either the A-only or the V-only condition, which are considered independent processing channels. The probability of word recognition in the presence of the two independent, non-interacting, modalities is given by:

(6) $$P_{s\,u\,m}=1-P_{f\,a\,i\,l}=P_{A}+P_{V}-P_{A}\times P_{V}$$

where *P*_*sum*_ is the probability to successfully recognize a word according to the summation model, *P*_*A*_ is the probability to recognize a word in the A-only condition, and *P*_*V*_ is the probability of recognizing a word in the V-only condition. Both *P*_*A*_ and *P*_*V*_ were estimated according to equations 1 and 2, but there were no additional free parameters to fit for the probability summation model. In order to demonstrate how well this model performs for various unimodal stimulus strengths, we split the data in four groups (Figure 6), as a first, simple approximation, consisting of poor or good V-only lipreading or average A-only listening accuracy (estimated recognition rate below or above 0.55, respectively; for A-only, recognition rates are averaged across SNR; as shown in Figures 2B, 3F–J). Note that there is a weak, negative correlation between the speech listening threshold and lipreading recognition rate at the word level; *r* = −0.39, 95%-HDI = −0.63 to −0.15, so that each group contains a slightly different number of subject-word combinations.

Despite the differences in unimodal performance, the best-fit performance curves (according to equations 1–3) for each of those groups followed a similar pattern. Auditory performance (Figure 6 – blue) degrades as the signal-to-noise ratio decreases; degradation is worse for words with poor auditory thresholds (Figures 6A,C). Visual performance (Figure 6 – red) is better than auditory performance for a larger range of SNRs if the visual word recognition rate is better (Figures 6A,B). Notably, for all groups, audiovisual performance (Figure 6 – green) is never worse than auditory performance; a clear audiovisual enhancement relative to auditory performance alone is present for a large range of SNRs. While audiovisual performance is typically also better than visual performance, at very low acoustic SNRs, the multisensory performance tends to be worse than lipreading performance (Figure 6, the green curves and circles drop below the red lines and circles). Overall, the fits to equations 1–3 followed the average correct scores nicely, although the AV fit (green) slightly under- and overshot the correct score at the lowest SNR for the high-accuracy and low-accuracy V-only data, respectively. The V-only fit (red) indicated slightly better performance than the average correct score for low-accuracy V-only data (Figures 6C,D).

Notably, the benchmark probability summation model can describe the audiovisual data quite well, at least qualitatively (Figure 6 – black). This model exhibits unimodal-like performance whenever either unimodal recognition abilities vastly outperforms the other, and shows maximum enhancement when the visual and auditory performances are equal.

We also fitted two other models that can exhibit (supra-additive) enhancements in audiovisual speech perception (Rouger et al., 2007; Ma et al., 2009). While qualitatively similar, our version of these models (that also include word and subject variability in the model parameters) performed worse than the probability-summation model (both in terms of how well the fit curves approximated the correct scores, and in terms of the BIC). We will not elaborate on these models here, but would like to note that neither these two models nor the probability-summation model allow for audiovisual performance to drop below visual performance.

### Inverse Effectiveness – Noise Level

**FIGURE 7:**
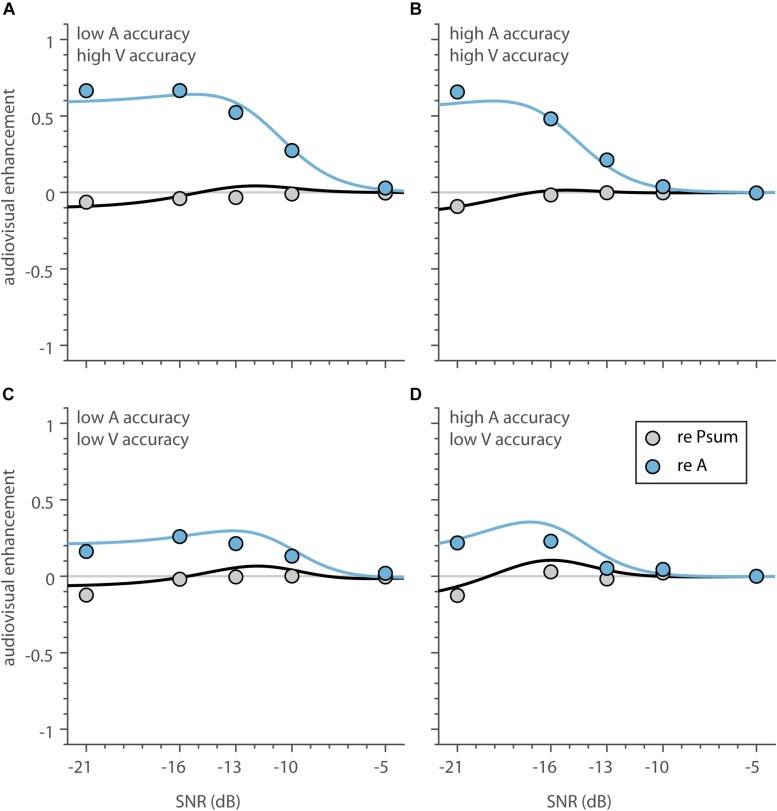
Audiovisual enhancement as a function of SNR. **(A–D)** The average audiovisual enhancement, expressed as proportion correct, as a function of SNR, compared to speech listening only (blue) and the proportion summation model (black). Curves (circles) indicate the enhancement calculated from the average model estimate (average correct score).

To test whether the multisensory data adhered to the principle of inverse effectiveness, we first determined the influence of SNR, as a measure of auditory stimulus intensity, on the magnitude of the audiovisual enhancement. For this purpose, we determined the audiovisual enhancement as the difference between the average audiovisual and auditory model fits and correct scores (Figure 6, green and blue, curves and circles). The shape of audiovisual enhancement is largely similar across the four groups (Figure 7, blue), and indicates (1) that auditory recognition performance improves by adding the visual information especially for low SNRs, and (2) the highest enhancement occurs at high to intermediate noise levels (SNR between −13 and −20 dB). For the lowest SNR of −21 dB, enhancement saturates or decreases slightly (for the correct scores only when A-only and V-only accuracy is low in Figure 7C). So, the principle of inverse effectiveness seems to apply to a large extent, when auditory SNR is considered as the measure of unimodal reliability.

We can also express the audiovisual enhancement relative to the benchmark model of statistical summation. For all 4 groups, the probability-summation model resembles AV speech recognition quite well (Figure 7; black lines close to 0). However, there is a slight deterioration at the lowest SNRs (maximum deterioration of −0.04 to −0.10 at an SNR of −21 dB).

### Inverse Effectiveness – Word and Subject Accuracy

Finally, we tested whether multisensory enhancement correlates negatively with unisensory responsiveness (i.e., A-only thresholds, V-only word and subject recognition rates; rather than stimulus intensity, i.e., SNR), as predicted by the principle of inverse effectiveness. To that end, we determined the multisensory enhancement as the difference in correct scores between the audiovisual and either the auditory, *E*_*AV–A*_, or visual, *E*_*AV–V*_, stimulus, for every word, subject and SNR combination. The slope of the relationship between multisensory enhancement and auditory thresholds or visual recognition rates, respectively, was determined through multiple linear regression analysis:

(7) $$\left\{ \begin{array}{c} E_{A\,V-A}=\beta_{0}-\beta_{1}\,\theta_{A}+\beta_{2}\,\rho_{V,w}+\beta_{3}\,\rho_{V,s}\\ E_{A\,V-V}=\beta_{4}-\beta_{5}\,\theta_{A}+\beta_{6}\,\rho_{V,w}+\beta_{7}\,\rho_{V,s} \end{array}\right.$$

with β_*1*_ the parameter of interest to infer effectiveness of the auditory response, and β_*6*_ and β_*7*_ of the visual response for words and subjects. The other parameters are included to account for confounds such as the effect of the other modality (e.g., the audiovisual enhancement over the auditory response will be negligible if the visual response is minimal). These parameters are an offset to the intercept and reflect the type of integration as shown by the audiovisual data (i.e., super-additive, additive, sub-additive). Note that for the auditory thresholds, the signs are inverted. This ensures that a negative slope would actually indicate inverse effectiveness, even though higher thresholds indicate a worse response.

**FIGURE 8:**
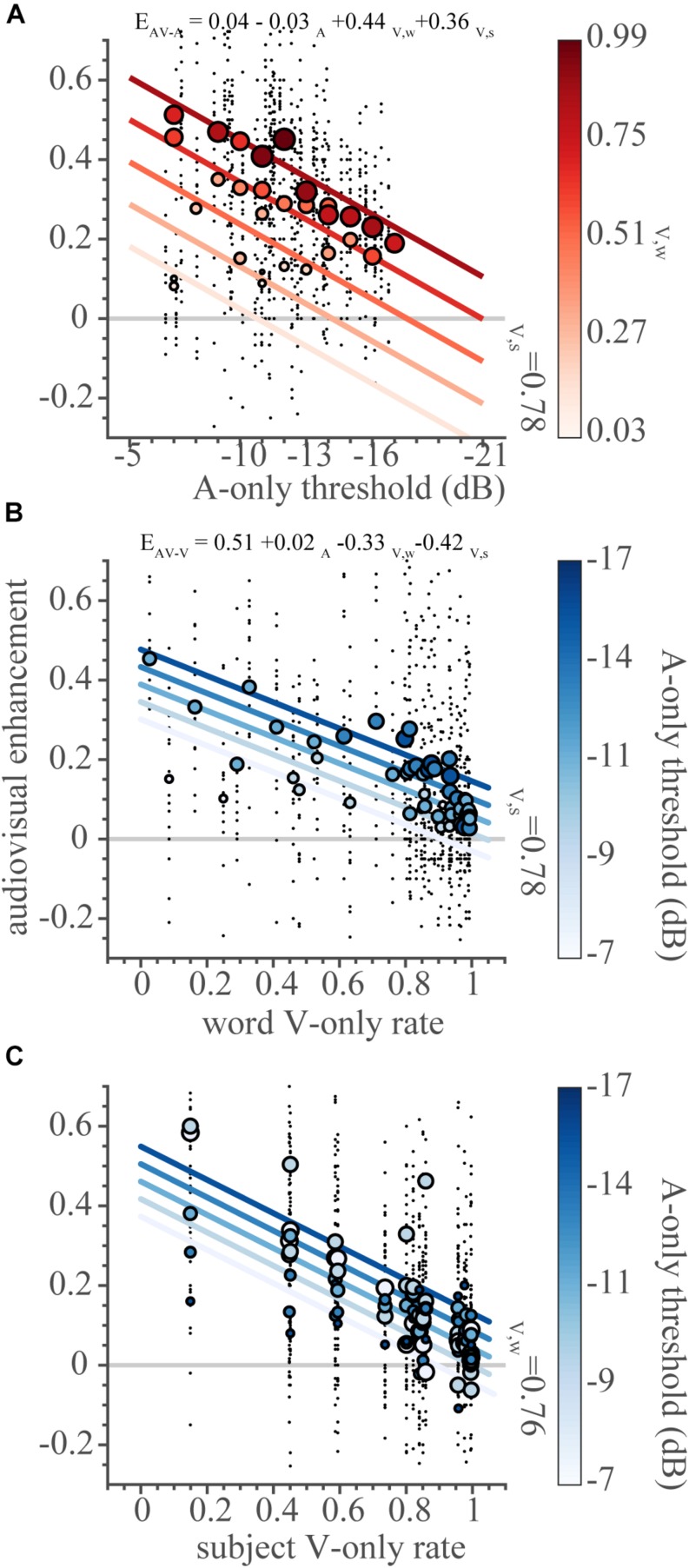
Inverse effectiveness. The audiovisual enhancement over unisensory responses (as defined in the text) as a function of the independent variables **(A)** auditory threshold, **(B)** visual word recognition rate, **(C)** visual subject recognition rate. Note that the *x*-axis is inverted in **(A)**. Black dots indicate the enhancement in correct score for every subject-word-SNR combination. To visualize the effects of the three independent variables on the dependent variable, we binned the variables as follows. The two-dimensional bins were centered on rounded threshold values and for five visual word recognition rates (from the minimum to the maximum rates in equidistant steps) in **(A)**, and on five auditory thresholds (from the minimum to the maximum thresholds in equidistant steps) and all visual word recognition rate values in **(B)** and visual subject recognition rates in **(C)**. Circles denote binned average correct scores. Lines indicate the best-fit multiple regression lines for the independent variable of interest (on the abscissa), with intercepts determined by the second, binned variable (indicated by the color bar) and the mean of the third variable (indicated by text). Dot size (color) denotes the cross-sensory performance level (as indicated by the color bars).

The audiovisual enhancement over the auditory response (*E*_*AV–A*_, Figure 8A) is larger for words with higher auditory thresholds, with an effectiveness slope β_1_ = −0.031 (95%-HDI: −0.035 to −0.027). The negative slope suggests that the auditory response to each word is inversely effective in driving the multisensory response. The magnitude of the enhancement over the auditory response increases when a word can be more easily recognized through lip-reading (i.e., high visual word recognition rate, dark filled dots). This is in line with the observation that the multisensory data follow probability summation quite well, reflecting an additive type of integration (Figures 6, 7). Importantly, the observed inverse effectiveness is not an artifact due to a ceiling effect, as the auditory response allowed for a larger performance benefit (Figure 8A, dotted line).

Multisensory enhancement over the visual response follows the same principles. Words with a low visual recognition rate were more effective at improving the AV response (Figure 8B), with an effectiveness slope β_6_ = −0.33 (95%-HDI: −0.38 to −0.29). Notably, even across subjects, the poorer lipreaders benefit more from audiovisual presentation than excellent lipreaders (Figure 8C), with an effectiveness slope β_7_ = −0.42 (95%-HDI: −0.46 to −0.38).

## Discussion

### Overview

This paper reports the occurrence of inverse effectiveness on the recognizability – visually or auditory - of individual words. We determined how well words presented in sentences can be recognized by normal-hearing subjects through listening and/or lipreading under noisy listening conditions. In line with previous research (Helfer, 1997; Grant and Seitz, 2000; Bernstein et al., 2004; Winn et al., 2013), we found that lipreading improves speech recognition by listening alone (Figures 5A, 6). However, we also observed that audiovisual performance levels fall below lipreading performance for the lowest SNR (Figures 5C, 6). Furthermore, we found that the improvements typically saturated at intermediate SNRs, which is largely in line with the principle of inverse effectiveness. We also observed inverse effectiveness across individual words and subjects (Figure 8): the data show that the benefit of adding cross-modal information increased when a word was poorly heard (Figure 8A), when a word was poorly seen (Figure 8B), or when the subject was a poor lipreader (Figure 8C).

### Performance in Lipreading

Our data demonstrate considerable variability in lipreading performance (Figure 2), which has been reported and discussed earlier in the literature (Bernstein et al., 2000). The average performance levels from the current study are relatively high, especially considering that the normal-hearing subjects were not specifically trained to lipread. This is consistent with earlier findings on word and sentence recognition tasks (Bernstein et al., 2000), although more recent papers have reported lower values (Rouger et al., 2007; Ross et al., 2007; Ma et al., 2009). One possible explanation for the high lipreading performance might be the use of the closed-set speech-recognition task (i.e., a limited set of words used in a forced-choice behavioral task).

### Performance in Speech Listening

The auditory scores varied mainly across words; subjects could all recognize words through listening at an almost equal performance level (Figure 3). Since all participants had normal hearing, and could therefore be expected to understand speech equally well, the limited variability between subjects corroborated that expectation. The analysis of speech recognition performance in the auditory-only condition revealed speech reception thresholds of −12.1 dB, which is lower than the threshold of −8.4 dB obtained from the original version of the Dutch Matrix test (Houben et al., 2014).

### Models for Audiovisual Enhancement

The behavioral improvement of audiovisual speech perception can be modeled in various ways. Typically, AV data are compared to the benchmark probability-summation model, in which the auditory and visual channels are considered independent, without true multisensory neural interactions. This model (Equation 6) matched the data closely (Figures 5, 6).

Rouger et al. (2007) found that an alternative, optimal-integration model could better describe their data. In their model, spectral-temporal audiovisual cues merge across modalities to optimize the amount of information required for word recognition. Our audiovisual data in poor lipreading conditions (i.e., visual recognition rate for a word is lower than 0.55) compares quite well to the speech-recognition abilities of the normal-hearing subjects of Rouger et al. (2007 – their Figure 3D) in the presence of a masking noise.

A third model was proposed by Ma et al. (2009), in which words were regarded as points in a multidimensional space, and word recognition becomes a probabilistic inference process. This Bayesian model assumes that certain words occur more frequently than other words (and are more easily recognized), and it uses this pre-knowledge (i.e., priors) to explain the recognition scores for all words.

It is hard to reconcile any of the three models with our observation that in low-SNR conditions, multisensory speech recognition is actually degraded compared to unimodal lipreading without accounting for non-stimulus factors affecting audiovisual speech recognition (Figures 4C, 5). The aforementioned models do not include a mechanism for divided attention between the two modalities (Bonnel and Hafter, 1998; Alsius et al., 2005). In such a scheme, the two separate information streams could actually lead to impaired performance in conditions in which either of the two signals may be ambiguous or weak. Thus, even though lipreading might provide sufficient information to recognize words, people are not able to divert their attention away from the auditory stream, despite the absence of a potential signal in that information stream.

### Inverse Effectiveness

We tested whether the principle of inverse effectiveness also holds in audiovisual speech recognition by: (i) modulating the acoustic signals re background noise, (ii) by investigating each subject’s lipreading ability, and (iii) by comparing to auditory and/or visual recognizability of words.

First, in line with several laboratory studies of multisensory integration using simple sensory stimuli (e.g., white noise bursts and LED flashes) (Meredith and Stein, 1986; Frens et al., 1995; Wallace et al., 1998; Corneil et al., 2002; Alais and Burr, 2004; Wallace et al., 2004; Bell et al., 2005; Körding et al., 2007; van Wanrooij et al., 2009; van Barneveld and van Wanrooij, 2013), a lower auditory SNR typically induced stronger multisensory enhancement. However, here we report that for the lowest SNRs (−21 dB) the enhancement saturated, or even slightly dropped (Figure 7C). This differs quantitatively with the data from Ma et al. (2009), who found a significant enhancement drop for low SNRs. Notably, however, Bayesian modeling of audiovisual enhancement in the study by Ma et al. (2009) suggested that the largest enhancement shifted to lower SNRs with decreasing vocabulary size. As the vocabulary size in the current experiment was limited to only 50 words (with only 10 possible choices per word category), the model by Ma et al. (2009) would also predict the largest enhancement at the lowest SNRs.

Secondly, evidence for inverse effectiveness can be found for individual lipreading abilities; worse lipreaders benefited more from the additional auditory information for the audiovisually presented sentences (Figure 8C). Finally, inverse effectiveness also plays a role at word-level performance, both for vision and for hearing: the hardest to-recognize words exhibited the strongest audiovisual enhancements relative to the unimodal condition (Figure 8). As such, this type of inverse effectiveness found is in line with basic multisensory integration results from earlier studies using stimuli with low-level features (simple noise bursts and LED flashes) and for studies using slightly more complex, spectro-temporally modulating stimuli (Bremen et al., 2017), but likely also involves a wide network of high-level feature processing (features such as word frequency, familiarity, audiovisual co-occurrence, task constraints; see also the limitation of this study in determining these effects in the following section).

### Matrix Test

The audiovisual speech material is based on an existing auditory-only matrix sentence test for Dutch native speakers (Houben et al., 2014; Houben and Dreschler, 2015). It is not immediately clear whether the observed results hold specifically for the Dutch language, or whether it is immaterial for which language this test has been developed. Numerous audiovisual speech recognition tests have been developed for the English language (Sumby and Pollack, 1954; MacLeod and Summerfield, 1990; Bernstein et al., 2004; Ross et al., 2007; Ma et al., 2009; Stevenson et al., 2015), with exceptions for native French (Rouger et al., 2007; Anderson Gosselin and Gagné, 2011) and Dutch speakers (Middelweerd and Plomp, 1987). Detailed comparisons are difficult also because the stimuli (monosyllables vs. words vs. sentences) and the subject populations (normal-hearing vs. hearing-impaired) differ. The use of a standardized test, such as the Matrix test, might facilitate comparisons, especially between normal-hearing and hearing-impaired listeners, since the Matrix test is also well-suited to test the hearing-impaired. Comparisons across languages might still be difficult, as, even though an auditory Matrix test is available in many languages (Hagerman, 1982; Ozimek et al., 2010; Hochmuth et al., 2012; Houben et al., 2014), the words may vary in their spectro-temporal properties and thresholds between languages.

Note that the use of this standardized Matrix test, that was constructed with the intention to evaluate hearing-impaired, includes words that are quite common and that are familiar to the subjects. The dependence of word recognition on higher-level factors beyond the low-level processing of spectro-temporal or articulatory stimulus representation is therefore hard – if not impossible – to determine with these speech materials.

## Conclusion

To conclude, lipreading enhances speech recognition (in line with earlier studies); this visual enhancement, however, is affected by the acoustic properties of the audiovisual scene. Visual enhancement for words that are easily recognized by vision alone is impoverished in high acoustic noise conditions. Audiovisual enhancements were highest for intermediate signal-to-noise ratios. Inverse effectiveness holds for words and subjects, for which the poorest visually/auditory-recognizable words underwent the strongest cross-modal enhancements.

## Materials and Methods

### Participants

Eighteen native Dutch-speaking adults (mean age = 26 years, range = 21–40) participated in this study. All gave their informed consent. They were screened for normal-hearing (within 20 dB HL range 0.5 – 8 kHz), and had normal or corrected-to-normal vision (see also Holmes, 2009; Stein et al., 2009 for a discussion on quantifying inverse effectiveness).

### Audiovisual Material

The speech material was based on the Dutch version of the speech-in-noise matrix test developed by Houben et al. (2014) in analogy to a Swedish test (Hagerman, 1982). In general, a matrix test uses complete sentences that are composed from a fixed matrix of words (Table 1). All created sentences shared the same grammatical structure (name, verb, numeral, adjective, object), but were semantically unpredictable. In principle, a set of 10^5^ different sentences could be created. Therefore, the test suffered little from potential training confounds when participants were tested multiple times. Houben et al. (2014) ensured that the occurrence of phonemes in their test was similar to standard Dutch. For the audiovisual version of the test reported here, we selected a subset of 180 (155 unique) sentences that were grouped into 9 lists of 20 sentences each. In every list, each of the 50 words from the matrix occurred twice, once in the first ten sentences and once in the second ten sentences.

The audio-video material was recorded in a sound-attenuated, semi-anechoic room, using an Olympus LS-5 audio recorder (24-bit/44.1 kHz sampling rate), and a Canon 60D video camera (1280 × 720, 720 p HD at 50 frames per second), respectively. All sentences were spoken by a Dutch female speech therapist. If a sentence was not articulated clearly, or if there was a sudden movement of the face or eyes, the sentence was re-recorded. The audio and video recordings were combined off-line using Final Cut Pro X (Mac App OS X Yosemite), and saved in MPEG-4 format, in H.264 codec.

### Experimental Setup

Audiovisual testing was carried out in the same room in which the material had been recorded. Stimulus presentation was controlled by a Dell PC (Dell Inc., Round Rock, TX, United States) running Matlab version 2014b (The Mathworks, Natick, MA, United States). Participants were seated at a table, 1.0 m in front of a PC screen (Dell LCD monitor, model: E2314Hf, Dell Inc., Round Rock, TX, United States). Sounds were played through an external PC sound card (Babyface, RME, Germany) and presented over one speaker (Control Model Series, model number: Control One, JBL, Los Angeles, CA, United States) placed 1.0 m in front of the participant, immediately above the screen (30° above the interaural plane). Speaker output was calibrated with an ISO-TECH Sound Level Meter (type SLM 1352P) at the position of the listener’s head, on the basis of the stationary masking noise.

### Stimuli

The stimuli contained digital video recordings of a female speaker reading aloud the sentences in Dutch (Figure 1). In the auditory-only presentation (A-only), the voice was presented without visual input (i.e., black screen, Figures 1A,C) with added background acoustic noise (Figure 1B). In the visual-only presentation (V-only) the video fragments of the female speaker were shown on the screen without an auditory speech signal and noise (Figure 1D). In the audiovisual presentation (AV), the video was presented with the corresponding auditory signal and the masking noise.

The masking noise was created following the procedure reported by Wagener et al. (2003). To that end, the 180 sentences were overlaid by applying a random circular shift. Repeating that procedure five times resulted in a stationary masking noise with the same spectral characteristics as the original speech material.

### Paradigm

All participants were tested in a closed-set speech-recognition test in A-only, V-only and AV conditions. Prior to the experiment, all participants familiarized themselves with the matrix of 50 words (10 words for each of the 5 categories, Table 1) and by practicing the task on 10 randomly selected AV sentences. No improvement in speech recognition was observed during the experimental sessions, which indicates that there was no recognition effect of procedural learning.

The masking noise started and ended 500 ms before and after the sentence presentation. The noise onset and offset included 250 ms (sin^2^, cos^2^) ramps. In the A-only and AV conditions, the masking noise was fixed at 65 dB SPL (A-weighted), with the speech sound presented at 44, 49, 52, 55, or 60 dB SPL (A-weighted) to obtain signal-to-noise ratios (SNRs) of −21, −16, −13, −10, and −5 dB, respectively. After presentation of the sentence and the end of the noise, the matrix of 50 words was shown on the screen (Table 1). Participants were instructed to choose one word from each of the 5 categories (10-alternative forced-choice task). Participants initiated the next trial by pressing the mouse-button.

For each of the sensory modalities (A-only, V-only, and AV), participants were tested in separate sessions on different days. In this way, fatigue and repetitive stimulus presentation were avoided. In each session, the nine lists of 20 sentences were presented. In the A-only and AV sessions, each sentence was assigned one of the five SNRs pseudo-randomly (each SNR was presented equally often as the others, i.e., 36 times in each session).

### Data Analysis

For every word (*w* = 1:50), subject (*s* = 1:18), SNR (*n* = 1:5) and sensory modality (*m* = 1:3), we determined the correct score, defined as the number of correct responses, z, divided by the number of presentations, N. The correct score, P(correct), is binomially distributed, in which the probability of a success is given by:

(8) P⁢(correct)∼Binomial⁢((1-γ)×F⁢(ψ)+γ,N)

where *F*(ψ) is a function that characterizes the recognition performance for the particular stimulus and subject parameters (subject parameters such as SNR and visual recognition rate), described by ψ; *γ* is the probability that the subject gives the correct answer, irrespective of the stimulus (the ‘guess rate’); (1-*γ*)*F*(ψ) + *γ* is the probability of success; N is the number of trials; and Binomial denotes the binomial distribution. Here, *γ* was set to 10% (0.1), as there were ten word alternatives per category. We estimated model parameters ψ, e.g., the recognition rates, *ρ* (i.e., how often words were recognized correctly at a given SNR) and the recognition thresholds, *θ* (i.e., the SNR at which words were recognized in 50% of the presentations), as described in the section “Results” (equations 1–3).

#### Statistical Analysis

Parameter estimation of Equations 1–8 was performed using a Bayesian statistical analysis. This analysis requires the definition of priors over the parameters. As a prior for the auditory thresholds, we chose the Gaussian distribution with mean 0 and standard deviation 100, and for the visual recognition rates we took a positive-only beta distribution, for which both shape parameters were set to 1. The audiovisual rate differences (Equation 5) were modeled as Gaussian distributions with the rates transformed to probit scale (see e.g., Lee and Wagenmakers, 2014, Chapter 9.3). For the multiple linear regression (equation 7; Kruschke, 2015), the data was modeled according to a *t*-distribution. For the priors on the parameters, Gaussian distributions with a mean of 0 and a standard deviation of 2 were chosen, after normalization of the data.

The estimation procedure relied on Markov Chain Monte Carlo (MCMC) techniques. The estimation algorithms were implemented in JAGS (Plummer, 2003) through matJAGS (Turner et al., 2013). Three MCMC chains of 10,000 samples were generated. The first 10,000 samples were discarded as burn-in. Convergence of the chains was determined visually, by checking that the shrink factor - R < 1.1 (Brooks and Gelman, 1998; Gelman et al., 2013), and by checking that the effective sample size > 1000 (Kass et al., 1998).

From these samples of the posterior distributions, we determined the mean and the 95% highest density interval (95%-HDI) as a centroid and uncertainty estimate of the parameters, respectively.

#### Model Selection

To test for the appropriateness of the models in equations 1–3, we compared them against less-restrictive models, including fully independent models. To that end, we determined the BIC for the models:

(9) $$\text{BIC}=\ln\left(n\right)\,k-2\,\ln\left(\hat{L}\right)$$

where *k* denotes the number of parameters of the model (e.g., 68 for equation 1 and 900 for a fully independent V-only model), *n* the number of samples (e.g., 900 for the V-only data), and $\hat{L}$ the maximized value of the binomial likelihood function (e.g., for those ρ_V,w_, and ρ_V,s_ that maximize the likelihood function for the V-only data at hand). The model with the lowest BIC is the preferred model. An alternative model-selection criterion, the Akaike Information Criterion (which contains a smaller penalty term for the number of parameters) yielded the same model selections.

## Data Availability Statement

All data are available from the Donders Institute for Brain, Cognition and Behavior repository at: http://hdl.handle.net/11633/aacawqmr.

## Ethics Statement

The experiments were carried out in accordance with the relevant institutional and national regulations and with the World Medical Association Declaration of Helsinki as revised in March 2017 (https://www.wma.net/policies-post/wma-declaration-of-helsinki-ethical-principles-for-medical-research-involving-human-subjects). The experiments were approved by the Ethics Committee of Arnhem-Nijmegen (project number NL24364.091.08, October 18, 2011). Written informed consent was obtained before conducting each experiment.

## Author Contributions

LR, MW, AO, EM, and AR designed the research and wrote the manuscript. LR performed the research. MW wrote the software. LR and MW analyzed the data.

## Conflict of Interest

The authors declare that the research was conducted in the absence of any commercial or financial relationships that could be construed as a potential conflict of interest.
